# Histone demethylase KDM4D inhibition suppresses renal cancer progression and angiogenesis through JAG1 signaling

**DOI:** 10.1038/s41420-021-00682-y

**Published:** 2021-10-11

**Authors:** Hao Yan, Liangsong Zhu, Jin Zhang, Zongming Lin

**Affiliations:** 1 Department of Urology, Zhongshan Hospital, Fudan University, Shanghai, China; 2 Department of Urology, Renji hospital, Shanghai Jiaotong University, Shanghai, China

**Keywords:** Tumour angiogenesis, Renal cell carcinoma

## Abstract

Kidney cancer, especially clear cell renal cell carcinoma (ccRCC), is one of the representative genitourinary tumors. Investigation of underlying mechanisms of ccRCC development is crucial for patient management. Histone demethylase KDM4D has been reported to be responsible for development of a variety of cancers. However, the role of KDM4D in ccRCC progression is poorly understood. In our study, we performed immunohistochemistry analysis of tissue microarrays first, and results showed that high expression level of KDM4D is connected with advanced Fuhrman grade (*p* = 0.0118) and lower overall survival (*p* = 0.0020). Then, we revealed that KDM4D can prompt ccRCC development by interacting with genes related to vessel morphogenesis. Finally, we disclosed that KDM4D directly interacts with JAG1 promoter and advances tumor angiogenesis by upregulating VEGFR-3 and antagonizing notch signaling. The results of our study indicate that KDM4D would be a potential prognostic marker and therapeutic target for ccRCC patients.

## Introduction

Renal cell carcinoma (RCC) is one of the prevailing type of urinary malignancies both in men and women [1]. The incidence and mortality rate of RCC has been increasing in several consecutive years except for some highly developed countries, whereas its pathogenesis and precise mechanism remain unknown [2, 3]. Smoking, obesity, hypertension, and genetic factors are all related risk factors that contribute to the occurrence of RCC [3]. RCC is historically and genetically divided into several subtypes, including clear cell RCC (ccRCC), chromophobe RCC, and papillary RCC. However, ccRCC is the most frequently diagnosed subtype, accounting for about 75% of kidney cancers [4, 5].

For patients with localized ccRCC, satisfactory clinical outcomes can be achieved after nephrectomy or radical nephrectomy. But for metastatic ccRCC, surgery can only reduce tumor burden and improve the symptoms. Besides, chemotherapy and radiotherapy have very limited therapeutic effects on patients with metastatic ccRCC. Research reveals that the genesis of ccRCC is intrinsically tied to Von Hippel-Lindau (VHL) gene inactivation or deletion. Inactivated VHL leads to the accumulation of hypoxia-inducible factors (HIFs), and upregulation of its downstream products VEGF and other hypoxia reactive genes [6, 7]. The in-depth understanding of the VHL–HIF–VEGF pathway has greatly accelerated the process of targeted therapy for kidney cancer. Studies have also confirmed that the mammalian target of rapamycin (mTOR) pathway is related to the genesis and progression of kidney cancer [8, 9], which paves the way for new drugs for ccRCC. At present, target therapy options for ccRCC includes the tyrosine kinase inhibitors (TKI) like sunitinib [10], mTOR inhibitors like tesirolimus which can directly interfere with it by acting on mTOR, thereby inhibiting tumor cell proliferation, transformation, and tumor angiogenesis [11], monoclonal antibody like bevacizumab which binds to circulating VEGF to prevent it from activating VEGFR, so as to achieve the purpose of anti-tumor angiogenesis [12]. However, patients in mounting numbers who treated with targeted drugs will inevitably develop drug resistance [13, 14]. Therefore, we are in dire need of novel therapeutic targets.

Recently, epigenetic modification has become one of the research hotspots [15]. As a major epigenetic alteration, histone methylation is a reversible process regulated by histone methyltransferase and demethylase [16]. Histone demethylases play a very important role in the expression of specific genes. Its mutation, abnormal expression or malfunction can cause cell development disorders and eventually lead to cancer initiation and progression. As a representative group of histone demethylases, KDM4 proteins which belongs to the JMJD (Jumonji C domain-containing) family have been identified that are associated with cancer proliferation, migration, invasion, and progression [16]. Latest research has shown that KDM4D, the last member of this family, is overexpressed in liver cancer and colon cancer [17, 18]. Hu et al. have found that inhibition of KDM4D can significantly suppress the tumor growth and metastatic in colon cancer by transcriptionally activating HIF-1β promotor [17]. KDM4D is also reported to be capable of promoting liver cancer formation and development by antagonizing tumor suppressor gene p53 and activating Wnt/β-catenin signaling pathway [18].

Considering the extensive involvement of KDM4D in different cancers, we suppose that KDM4D may also act as a cancer driver gene in ccRCC. Therefore, the present study aims to investigate the expression of KDM4D in human ccRCC samples, and observes its involvement in the proliferation, apoptosis, and angiogenesis, thereby providing a new theoretical basis for the diagnosis and treatment of ccRCC.

## Results

### KDM4D is a potential predict marker for prognosis of ccRCC

**Fig. 1:**
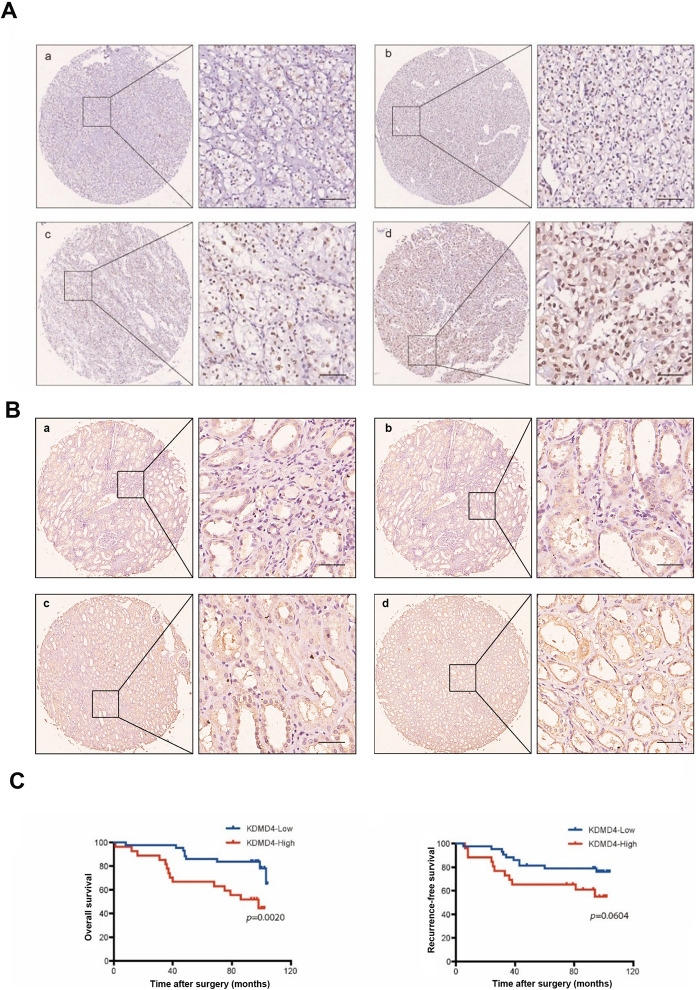
High expression level of KDM4D portend a worse prognosis and outcome. **A** Images of IHC staining of KDM4D in tissue microarrays. a minimum expression, b low expression, c moderate expression, d high expression. Scale bar = 100 μm. **B** Images of IHC staining of KDM4D in paracancerous tissue. a minimum expression, b low expression, c moderate expression, d high expression. Scale bar = 100 μm. **C** Kaplan–Meier curves of overall survival and recurrence-free survival, OS in KDM4D-high group is significantly lower than KDM4D-low group (*p* = 0.0020).

To reveal the expression of KDM4D in kidney cancer tissues, we performed immunohistochemical staining on tissue microarrays of 70 ccRCC patients with complete clinical data and follow-up information (as shown in Table 1). The patients are divided into two groups based on the expression level of KDM4D. After statistical analysis, we found that high expression of KDM4D was significantly associated with advanced Fuhrman grade (*p* = 0.0118, Table 1) and metastasis after surgery (*p* = 0.00475, Table 1). All the above results lead to a potential correlation between KDM4D and poor prognosis of ccRCC patients, so we used Kaplan–Meier survival analysis to compare the OS and PFS of patients in two groups. The results suggest that the high expression of KDM4D is negatively correlated with OS (*P* = 0.0020, Fig. 1B). But there is no significant difference in PFS between the two groups (*P* = 0.0604, Fig. 1C).

**Table 1:** Clinicopathological characteristics of ccRCC patients in relation to KDM4D expression level.

Characteristics	Patients		Tumor KDM4D expression		*p*-value
	*n*	%	Low	High	
All patients	70	100	43	27	
Gender					0.899
Male	55	78.6%	34	21	
Female	15	21.4%	9	6	
Age (years)					0.508
≤55	32	45.7%	21	11	
>55	38	54.3%	22	16	
TNM stage					0.256
I+II	68	97.1%	41	27	
III+IV	2	2.9%	2	0	
pT stage					0.256
T1 + T2	68	97.1%	41	27	
T3 + T4	2	2.9%	2	0	
pN stage					0
N0	70	100%	43	27	
N1	0	0	0	0	
Pm stage					0
M0	70	100%	43	27	
M1	0	0	0	0	
Furman grade					0.0118*
I + II	57	81.4%	39	18	
III + IV	13	18.6%	4	9	
Tumor size(cm)					0.606
≤4	39	55.7%	25	14	
>5	31	44.3%	18	13	
Metastasis for surgery					0.00475*
Yes	16	11.4%	5	11	
No	54	88.6%	38	16

### KDM4D facilitates the development of ccRCC cells

**Fig. 2:**
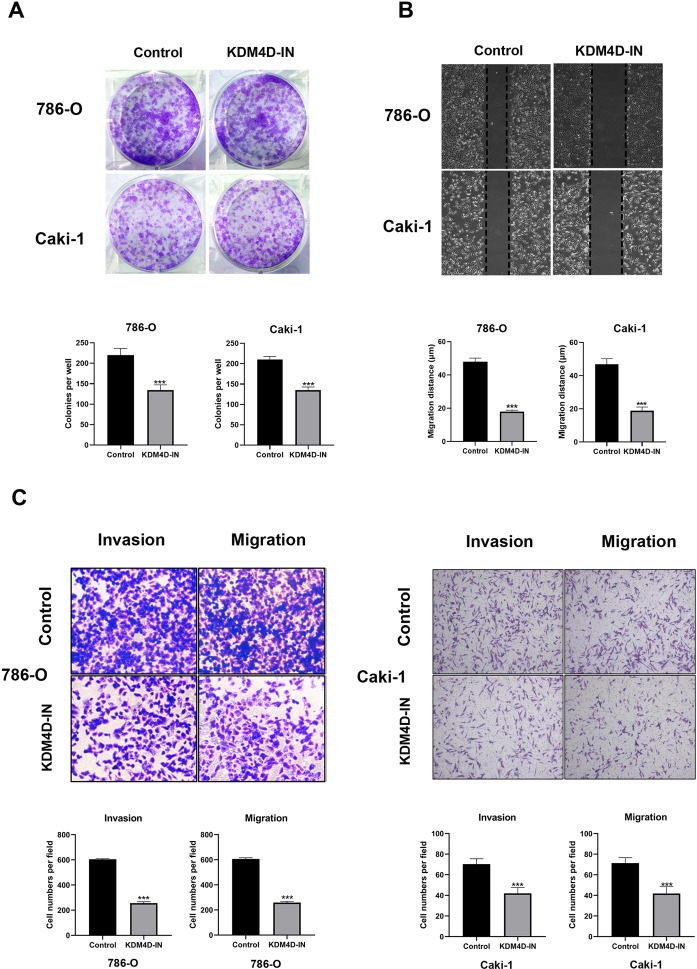
KDM4D inhibition reduces the colony formation, migration, and invasion ability of Caki-1 and 786-O cells. **A** Colony formation assays were performed to evaluate the effect of KDM4D inhibition on colony-forming capacity of ccRCC cells. Results have demonstrated that the ability of colony formation in both types of cells was significantly restrained by KDM4D inhibitor. ***p < 0.001. **B** The effects of KDM4D inhibition on cell migration in 786-O and Caki-1 cells were examined by wound healing assays. ***p < 0.001. **C** Images of transwell invasion and migration assays which were used to evaluate the influence of KDM4D inhibition on invasion and migration ability of renal cancer cells. The quantitative analysis was presented below.

We treated 786-O and Caki-1 cells with 0.5 μm KDM4D inhibitor (KDM4D-IN-1, MedChemExpress LLC, New Jersey, USA) and performed colony formation assays in 10 cm dish, wound healing assays in 6-well plate, invasion and migration assays in Transwell to evaluate the effect of KDM4D on ccRCC proliferation and development. As shown in Fig. 2A, the results demonstrated that KDM4D inhibition could significantly suppress the colony formation ability of 786-O cells and Caki-1 cells (*p* < 0.001). KDM4D inhibition can also minimize migration of ccRCC cells, as demonstrated in Fig. 2B, C, the wound healing ability of the cells in the control group was significantly higher than that of the KDM4D-IN group (*p* < 0.001, Fig. 2B), and KDM4D significantly promotes the migration of kidney cancer cells (*p* < 0.001, Fig. 2C). We also detect the effect of KDM4D inhibition on invasion ability of ccRCC cells, the results indicate that KDM4D dramatically facilitates invasion of ccRCC cells (*p* < 0.001, Fig. 2C). We also detected the changes in KDM4D expression in 786-O cells after using inhibitors, and the results showed that the expression level of KDM4D protein in 786-O cells was significantly reduced (*P* < 0.001, Supplementary Fig. 1A, B).

### Inhibition of KDM4D increases ccRCC cell apoptosis and suppresses proliferation

**Fig. 3:**
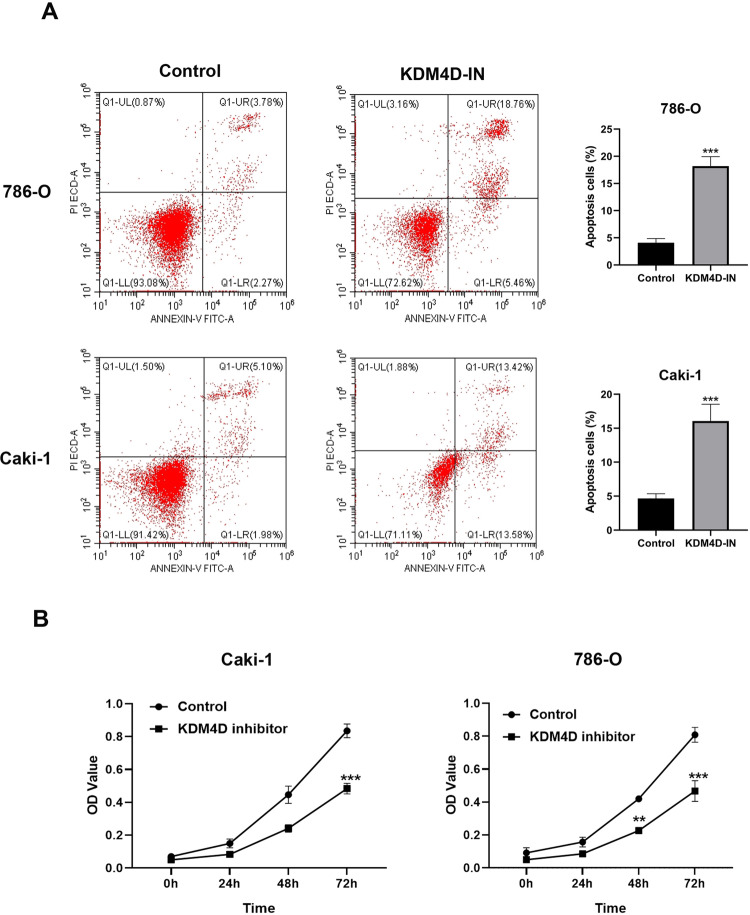
KDM4D inhibition suppress the proliferation of ccRCC cells and promote apoptosis. **A** Cell apoptosis assays demonstrate that KDM4D inhibition reduces tumor cell apoptosis. **B** The CCK8 assays showed that the proliferation of renal cancer cells was significantly inhibited by KDM4D inhibitor. **p < 0.05, ***p < 0.01.

To investigate the effect of KDM4D inhibition on the proliferation and apoptosis of renal cancer cells, we performed flow cytometry to detect apoptotic cells and CCK8 assays to assess the proliferation activity. As shown in Fig. 3A, KDM4D inhibition prominently augment apoptosis of ccRCC cells (*p* < 0.001). The results of CCK-8 assays illustrate that the proliferation of 786-O cells decreased significantly on 48 h in the KDM4D-IN group as compared with the control group (*p* < 0.05, Fig. 3B), and the differences between two groups increase further on 72 h both in 786-O cells and Caki-1 cells (*p* < 0.001).

### KDM4D promotes tumor angiogenesis by interacting with JAG1

**Fig. 4:**
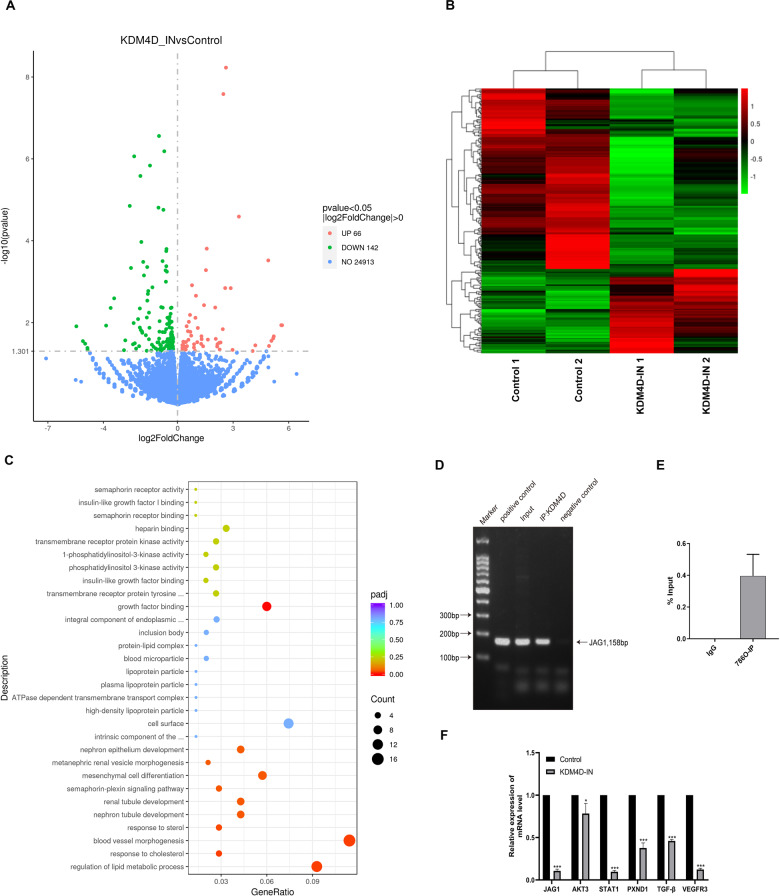
KDM4D promotes ccRCC development by transcriptionally activating JAG1. **A** Volcano map of the upregulated and downregulated genes. **B** Differentially expressed genes in renal cancer cells treated with or without KDM4D inhibitor are displayed by heat map. **C** KEGG analysis presents that blood vessel morphogenesis is the most enriched pathway. Related genes are listed in Supplementary Table 2. **D**, **E** The relative expression of downregulated mRNA involved in blood vessel morphogenesis. **F** The results of ChIP-qPCR assays showed that KDM4D could directly bind to the promoter of JAG1 in 786-O cells. ***p < 0.001.

To clarify the detailed mechanism of KDM4D in renal cancer cell development, we performed RNA-seq analysis for 786-O cells treated with KDM4D inhibitor or not. The differentially expressed genes are indicated in red for upregulated and green for downregulated as shown in the heatmap (Fig. 4A). Volcano map of the differentially expressed genes was shown in Fig. 4B. The enrichment of KEGG analysis showed genes from different pathways were downregulated in KDM4D-inhibited 786-O cells, especially blood vessel morphogenesis (Fig. 4C). Then we detected the mRNA expression of the genes indicated by the KEGG analysis, and results showed several genes especially JAG1 were significantly decreased in KDM4D-inhibited 786-O cells compared with the control group (Fig. 4D). By searching the GEPIA database, we also found that KDM4D was positively correlated with JAG1 (Supplementary Fig. 2). Then we assume that one possible mechanism of ccRCC progression was mediated by KDM4D through interaction with JAG1. To verify our presumption, we investigated the interaction between KDM4D protein and the JAG1 gene promoter by performing CHIP-qPCR assays. As shown in Fig. 4E, the target bands are clearly visible and detected in both the input and the KDM4D group. The signals obtained in CHIP were normalized by the percent input (Fig. 4F). The results of electrophoresis and qPCR certify that KDM4D can directly bind to the promoter region of JAG1.

### KDM4D inhibition reduces angiogenesis in ccRCC in vitro

**Fig. 5:**
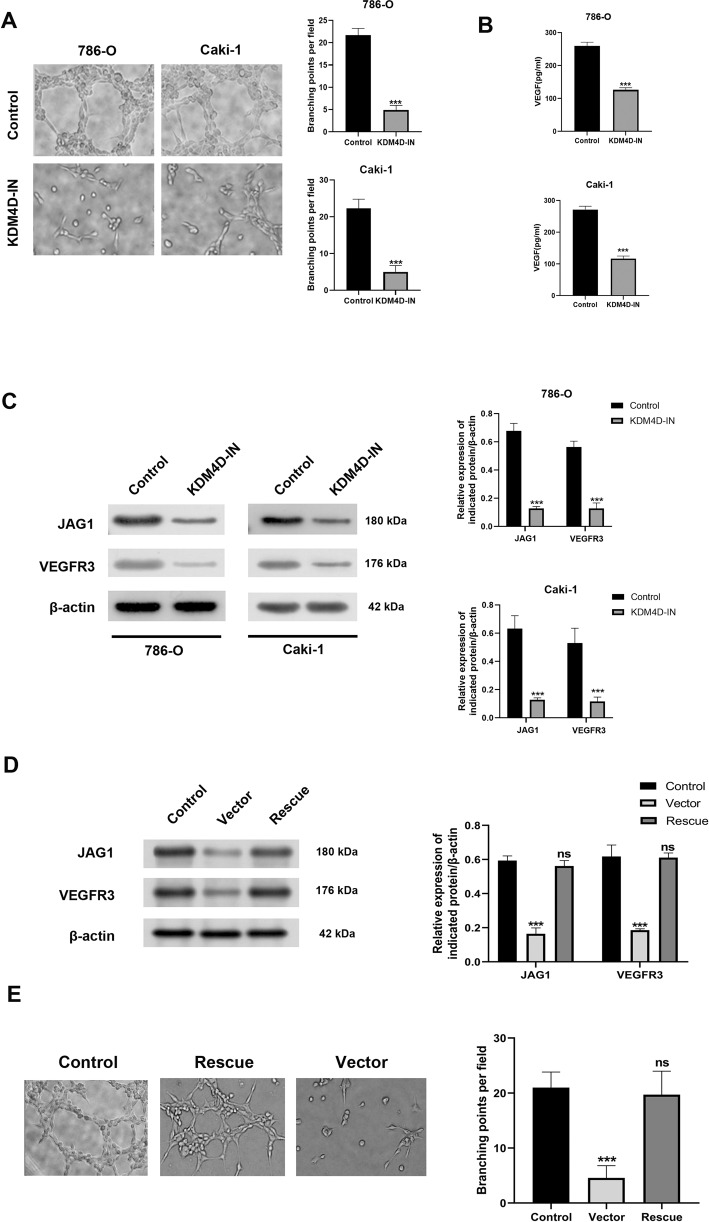
KDM4D promote angiogenesis of ccRCC cells in vitro. **A** KDM4D inhibition significantly retards the tube formation of HUVEC incubated in cell conditioned media. ***p < 0.001. **B** ELISA assays showed that KDM4D inhibition could reduce VEGF secretion in 786-O and Caki-1 cells. ***p < 0.001. **C** Western blot revealed that JAG1 expression was significantly decreased after inhibition of KDM4D in both types of renal cancer cells. **D** Western blots show that the expression of VEGFR-3 is elevated in JAG1-upregulated 786-O cells. ***p < 0.001. **E** JAG1 upregulation after KDM4D inhibition in 786-O cells restored the proangiogenic effect of KDM4D. ***p < 0.001.

To further observe the effect of KDM4D on tumor angiogenesis, we performed tube formation assays. As shown in Fig. 5A, we found that cells in control group immensely induced tube formation of HUVEC cells compared with KDM4D-IN group (*p* < 0.001). Results of ELISA assays have shown that VEGF secretion is markedly decreased after KDM4D inhibition (*p* < 0.001, Fig. 5B). In addition, results of Western blot demonstrated a notably decreased protein expression level of JAG1 and VEGFR-3 in KDM4D-inhibited ccRCC cells (*p* < 0.001, Fig. 5C). To further validate that KDM4D mainly promotes angiogenesis through JAG1, we conducted a rescue experiment by upregulating the JAG1 gene in 786-O cells after KDM4D inhibition, and found that after JAG1 was upregulated, the proangiogenic effect of KDM4D in 786-O has been significantly restored (*p* < 0.001, Fig. 5D, E and Supplementary Fig. 3). The above data suggest that KDM4D potentially influences angiogenesis by activating JAG1 in ccRCC cells.

### Inhibition of KDM4D suppresses ccRCC cell proliferation and angiogenesis in vivo

**Fig. 6:**
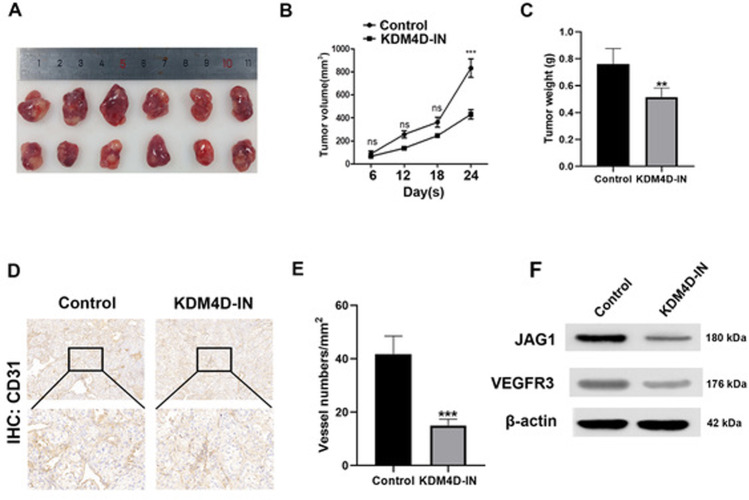
KDM4D inhibition suppresses tumor growth and angiogenesis in animal model. **A** Images of transplanted tumors excised from nude mice. **B** Tumor growth was significantly inhibited in KDM4D-IN group. **C** KDM4D inhibition tumors show a significant decrease in tumor weight. **p < 0.01 and ***p < 0.001. **D** Immunohistochemical staining was used to detect the expression of CD31 in two groups of transplanted tumors. **E** Quantitive analysis of vessel numbers based on the IHC staining of CD31. ***p < 0.001. **F** Images of Western blot bands demonstrate that JAG1 and VEGFR3 were downregulated due to the inhibition of KDM4D.

The effects of KDM4D on tumor angiogenesis in vivo were assessed by conducting xenograft tumor assays in nude mice. In contrast to the control group, we found that tumor in the KDM4D-IN group grew significantly slower (*p* < 0.001, Fig. 6B). In addition, the weight of tumors was significantly increased in the control group when compared with the KDM4D-IN group (*p* < 0.01, Fig. 6A, C). The vascularization status of the transplanted tumors was evaluated by IHC staining of CD31 in the two groups. As shown in Fig. 6D, E, CD31 expression was strikingly elevated in the control group compared with the KDM4D-IN group (*p* < 0.001, Fig. 6E), indicating that the process of angiogenesis in the KDM4D-IN group was attenuated because KDM4D was inhibited in 786-O cells. Western blot analysis of xenografts substantiated that the expression of JAG1 and VEGFR-3 was significantly decreased in KDM4D-IN group (*p* < 0.001, Fig. 6F). These results manifest that KDM4D is pivotal to tumor angiogenesis and proliferation of ccRCC.

## Discussion

Although the mechanism of RCC has been thoroughly investigated by researchers, we still need novel targets to break the glass ceiling of the management of RCC. Studies have shown that histone demethylase KDM4D participates in different types of tumors by interacting with specific genes [17–19]. However, the regulatory effect of KDM4D on ccRCC is relatively unexplored.

In our study, we first examined the relationship between KDM4D and the clinical outcomes of ccRCC patients. Results have shown that high KDM4D expression significantly correlates with a worse outcome of ccRCC patients. This indicates that post-translational epigenetic alterations are possibly involved in ccRCC progression and KDM4D may be a cancer driver gene of ccRCC. To further verify our conjecture, we treated the renal cancer cell lines Caki-1 and 786-O with KDM4D inhibitors, and then performed a series of experiments. As a result, we found that inhibiting KDM4D can restrain the ability of clonal formation, migration, and invasion of renal cancer cells. In an attempt to clarify the mechanisms of KDM4D on the development of ccRCC, we performed RNA-sequence analysis. The results of KEGG analysis potentially point to genes pertaining to blood vessel morphogenesis. Blood vessel morphogenesis is a complex process that is vital for tumor growth, progression, and metastasis [20]. Next, we evaluated the role of KDM4D in angiopoiesis of kidney cancer and found that KDM4D inhibition markedly impaired neovascularization in vitro and in vivo by HUVEC tube formation assay and immumohistochemical staining of CD31. To elucidate molecular mechanisms of angiogenesis induced by KDM4D, we detected the mRNA level of vasculogenesis-related genes that suggest by the RNA-sequence analysis. As expected, JAG1, PLXND1, TGF-β, and AKT3 were all downregulated after KDM4D inhibition. Among the genes above, JAG1 is widely reported to augment angiogenesis and tumor growth in animal model [21]. Hence, we hypothesized that KDM4D may promote the progression of ccRCC mainly through activating a Notch receptor ligand named JAG1. JAG1 is mainly expressed in stalk cells, a phenotype of epithelial cells (ECs). In the process of angiogenic growth, ECs will convert into two different phenotypes, tip cells which are responsible for initiating sprouting and stalk cells which is conducive to the stability of the emerging vessels. A concerted balance between the forthcoming sprouting and the maintenance of existing vessels is the prerequisite of angiogenesis. The Notch signaling is a pivotal pathway which controls the transition of ECs between tip and stalk cells. JAG1 and another notch ligand, Dll4, have opposite effects on this process [22]. Numerous studies have proved that after blocking Dll4, despite the increase in tumor angiogenesis, the structural defects and poor perfusion will aggravate the degree of ischemia and hypoxia of tumor cells, and effectively inhibited the growth of tumors [23–26]. Interestingly, JAG1 can counteract the effects of Dll4 on stalk cells, which promotes ECs proliferation and cytogenesis of tip cells dynamically. Importantly, the results of CHIP-qPCR assays certify that KDM4D interacts with the JAG1 promoter, which means KDM4D can transcriptionally activate JAG1 and promote ccRCC angiogenesis and development.

Notch activation was reported to downregulate the expression of VEGFR-2 and VEGFR-3, both of which were shown to have proangiogenic effects [27–29]. We observed that KDM4D inhibition in ccRCC significantly reduced VEGFR-3 expression. This may be due to the downregulation of JAG1 leading to the weakening of its antagonistic effect on Dll4, resulting in the downregulation of VEGFR-2 and VEGFR-3 expression. Furthermore, we investigate the effect of KDM4D on development and angiogenesis of kidney cancer cells in vivo. Results showed that inhibition of KDM4D can indeed impede the growth of xenograft and tumor angiogenesis. The above data suggested that KDM4D induces tumor angiogenesis mainly via JAG1 expression. On the one hand, KDM4D binds to the promoter of JAG1 and upregulates the expression level of VEGFR-3; on the other hand, activated JAG1 may counteract Dll4-Notch signaling pathway to prevent non-productive sprouting.

All in all, the research discusses that KDM4D predicts a worse prognosis and regulates the proliferation, migration, and angiogenesis of renal cancer. Therefore, KDM4D could be exploited as a prognostic marker or a target for anti-cancer therapy, but its specificity and practicality require rigorous preclinical testing in the future.

## Methods

### Tissue immunohistochemistry

The analysis of tissue microarrays (TMAs) which contains 70 cancerous and paracancerous tissues from patients with ccRCC was approved by the Ethics Board of Zhongshan Hospital, Fudan University. The pathological information of all samples was determined by two pathologists separately. The clinical characteristics were recorded and categorized based on a standardized system [30, 31]. Metastasis was assessed by imaging and laboratory tests. Immunohistochemistry (IHC) was performed to analyze the localization of KDM4D. The IHC score of KDM4D was calculated by two observers (Dr. Yan and Dr. Zhu) and the expression level of KDM4D was divided into two groups according to the IHC score as described before [32].

### Cell culture and inhibition of KDM4D

Human ccRCC cell line Caki-1 and adenocarcinoma cell line 786-O were used in this study (both were obtained from ATCC, Manassas, VA, USA). Cells were maintained in the RMPI-1640 medium (Thermo Fisher Scientific Inc., MA, USA) supplemented with 10% fetal bovine serum (FBS, Thermo Fisher Scientific Inc., MA, USA). Both the 786-O and Caki-1 cells were divided into two groups, the control group and the KDM4D-IN group. The KDM4D-IN group was treated with 0.5 μm KMD4D inhibitor (KDM4D-IN-1, MedChemExpress LLC, New Jersey, USA) for 24 h before being harvested for other experiments. For rescue experiment, 786-O cells inhibited by KDM4D inhibitor were transfected with pcDNA-3.1(+)-JAG1 plasmid and control vector plasmid. Cell cultures were maintained at 37 °C in a humidified atmosphere with 5% CO_2_ and medium was replaced when necessary.

### Colony formation assay

After being digested by trypsin, the cells treated by KDM4D inhibitor or not were resuspended in RPMI-1640 medium and seeded on a 6-well plate at a density of 500 cells per well. The culture was maintained in RPMI-1640 medium and terminated when visible clones appeared. Next, the cells were harvested and fixed with 4% paraformaldehyde for 15 min. The cells were then stained with crystal violet and dried at room temperature. Finally, the number of clones which contains more than 50 cells were counted under the microscope.

### Tumor mirgration and invasion assays

Caki-1 cells and 786-O cells were trypsinized, resuspended in serum-free culture medium, and counted. For migration assays, 2.5 × 10^5^ cells were seeded in the upper membrane of each transwell chamber. For invasion assays, the upper chamber membranes of transwell were evenly covered with Matrigel (BD science, New Jersey, USA) and incubated for 1 h before the cells were seeded. After 24 h of conventional culture, cells in the upper chamber were gently wiped off by a cotton swab. Each chamber was fixed with methanol, stained with crystal violet, washed with PBS, and dried at room temperature. Six fields were randomly selected to take pictures under a microscope.

### Wound-healing assays

Cells were harvested and seeded on six-well plates with 5 × 10^5^ per well. To simulate a wound, the cell surface was scratched by a 10-μl sterile plastic pipette tip with a straightedge when cells reached a confluent of 85 to 90%. Then the cells were cultured in serum-free medium and incubated at 37 °C. Overnight, wound images were collected under a microscope. The distance of the wound was measured and recorded, and the migration distance of cells in each group was calculated.

### Quantitative real-time PCR

Cells treated with or without KDM4D inhibitor were prepared for RNA extraction. Briefly, RNA was extracted by TRIpure total RNA extraction reagent (ELK Biotechnology, Wuhan, China). EntiLink™ 1st Strand cDNA Synthesis Kit (ELK Biotechnology, Wuhan, China) was used to synthesize cDNA according to the manufacturer’s instructions. Quantitative real-time PCR was performed using the StepOne™ Real-Time PCR (Life technologies, Wuhan, China) with specific primers (sequences were shown in Supplementary Table 1). The relative expression of RNAs was calculated using 2 − ΔΔCt method.

### Western blot

Proteins were collected from cell and tissues using the RIPA lysis buffer (Thermo Fisher Scientific Inc., MA, USA). The buffer was dissolved and mixed with protease and phosphatase inhibitors before being added into cells or tissues. Then proteins were separated by sodium dodecyl sulfate polyacrylamide gel electrophoresis (SDS-PAGE) and transferred to polyvinylidene fluoride (PVDF) membranes. After transfer, membranes were put into 5% bovine serum albumin and sealed at 25 °C for 2 h. Next, membranes were washed thrice with tris buffered saline tween (TBST) on a shaker for 5 min each time and incubated with TBST diluted primary antibody including KDM4D (ab93694, Abcam, Cambridge, MA, USA), JAG1 (ab109536, Abcam, Cambridge, MA, USA), VEGFR3 (ab243232, Abcam, Cambridge, MA, USA) and β-actin (ab8227, Abcam, Cambridge, MA, USA) at 4 °C. Overnight, membranes were washed by TBST and incubated with horseradish peroxidase (HRP) labeled secondary antibody (Jackson 1:2000) for 2 h at 25 °C. Finally, membranes were reacted with electrochemical luminescence regents (Thermo Fisher Scientific Inc., MA, USA) and photographed in dark room.

### Cell viability assay

The proliferation of kidney cancer cells was determined by CCK-8 assays (Dojindo Molecular Technologies, Kumamoto, Japan). Generally, renal cancer cells were seeded into 96-well plates and incubated for 0, 24, 48, and 72 h. Then the medium in each well was discarded and replaced by 100 μl of serum-free medium mixed with 10 μl of CCK-8 under dark condition. After incubated for 2 h, OD values at the absorbance of 470 nm were recorded.

### Cell apoptosis assay

For cell apoptosis analysis, cells were collected and centrifuged at 1000 *g* for 5 min. After the supernatant was discarded, the cells were counted and gently resuspended in phosphate buffered saline (PBS). Reagents were added according to the protocol of Annexin/PI kit (Beyotime, Shanghai, China). After incubating at room temperature (20–25 °C) for 10–20 min in the dark, cells were collected and detected by FACS Canto II (BD science, New Jersey, USA).

### Endothelial cell tube formation assay

Both Caki-1 and 786-O cells were treated overnight in RPMI-1640 medium supplemented without serum for 24 h to obtain the cell-conditioned medium. Then, 5000 HUVECs cells were seeded on μ-Slide Angiogenesis (ibidi, Gräfelfing, Germany) covered by Matrigel (BD science, New Jersey, USA) and incubated with 786-O or Caki-1 cell-conditioned media. Images of tube formation were captured under a microscope, and the number of branches was quantified.

### Enzyme-linked immunosorbent assays (ELISA)

Renal cancer cells treated with/without KDM4D inhibitor were cultured in FBS-free RPMI-1640 medium overnight. Then, the conditioned medium was collected for VEGF analysis using the Human VEGF ELISA kit (Beyotime, Shanghai, China). The OD values were obtained based on absorbance at 470 nm.

### Chromatin immunoprecipitation (ChIP) assay

Chromatin immunoprecipitation assays were conducted using a SimpleChIP Plus Enzymatic Chromatin IP Kit (9005 S, CST, Boston, Massachusetts, USA). The sense primer for JAG1 was 5′-CTGAGCAACGATCCCTTCCA-3′, the antisense primer for JAG1 was 5′-GGGTAGTGCGAGGAGGAACT-3′. The positive control used in the study is Histone H3 antibody (D2B12, CST, Boston, Massachusetts, USA).

### Subcutaneous xenotransplanted tumor model

All animal experiments were approved by the Animal Care and Use Committee of Zhongshan Hospital Fudan University (Shanghai, China). Twelve 8-week-old female athymic nude mice were divided into two groups, the control group and the KDM4D-IN group, and tumor were transplanted by injection of 2 × 10^6^ 786-O cells resuspended in a solution of equal parts Matrigel (BD science, New Jersey, USA) and PBS to the subcutaneous tissue of nude mice. KDM4D inhibitor (KDM4D-IN-1, MedChemExpress LLC, New Jersey, USA) was diluted by 90% sterile water and 10% DMSO, and intraperitoneally injected for 3 weeks in the KDM4D-IN group. The weights of mice were measured twice a week. Xenografts were removed eventually and submerged in 4% paraformaldehyde and embedded by paraffin for IHC analysis. In brief, excised xenografts were sliced into 5 μm sections and stained with antibodies against CD31 (ab28364, Abcam, Cambridge, MA, USA). Six area were randomly chosen in each section under the microscope. The vessel numbers and KDM4D expression were calculated by Image J1.50i (National Institutes of Health, MD, USA).

### Statistical analysis

Chi-squared test was used to evaluate the relationship between KDM4D and clinical characteristics. Kaplan–Meier method was used to plot the overall survival (OS) and progression-free survival (PFS) curves. Two-tailed, paired *t*-test or unpaired *t*-test analysis was performed to compare the significance between cells treated with/without KDM4D in different assays. All the tests were performed in GraphPad Prism 8.0.1 (GraphPad Software, San Diego, CA, USA) and *p* value <0.05 indicates statistically significant.

## Supplementary information

Supplementary materials

Supplementary materials

## Data Availability

The datasets generated during the current study are available from the corresponding author on reasonable request.
