# Supplementary material for: Histone demethylase KDM4D inhibition suppresses renal cancer progression and angiogenesis through JAG1 signaling
Source: Cell Death Discov. 2021 Oct 11;7:284. doi: 10.1038/s41420-021-00682-y (PMC8526739; doi:10.1038/s41420-021-00682-y)
Supplement: Supplementary file 1 — Supplementary materials [file 41420_2021_682_MOESM1_ESM.docx]

**Supplementary Figure 1 A** Western blot images of 786-O cells treated with or without
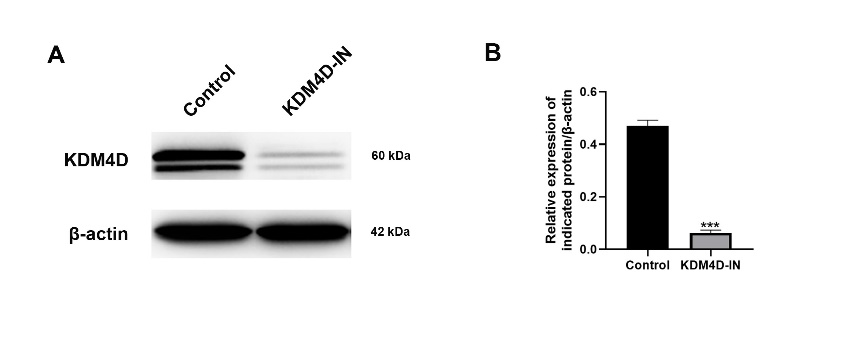
KDM4D inhibitor **B** The protein expression of KDM4D was significantly decreased in KDM4D-IN group. ***p < 0.001.


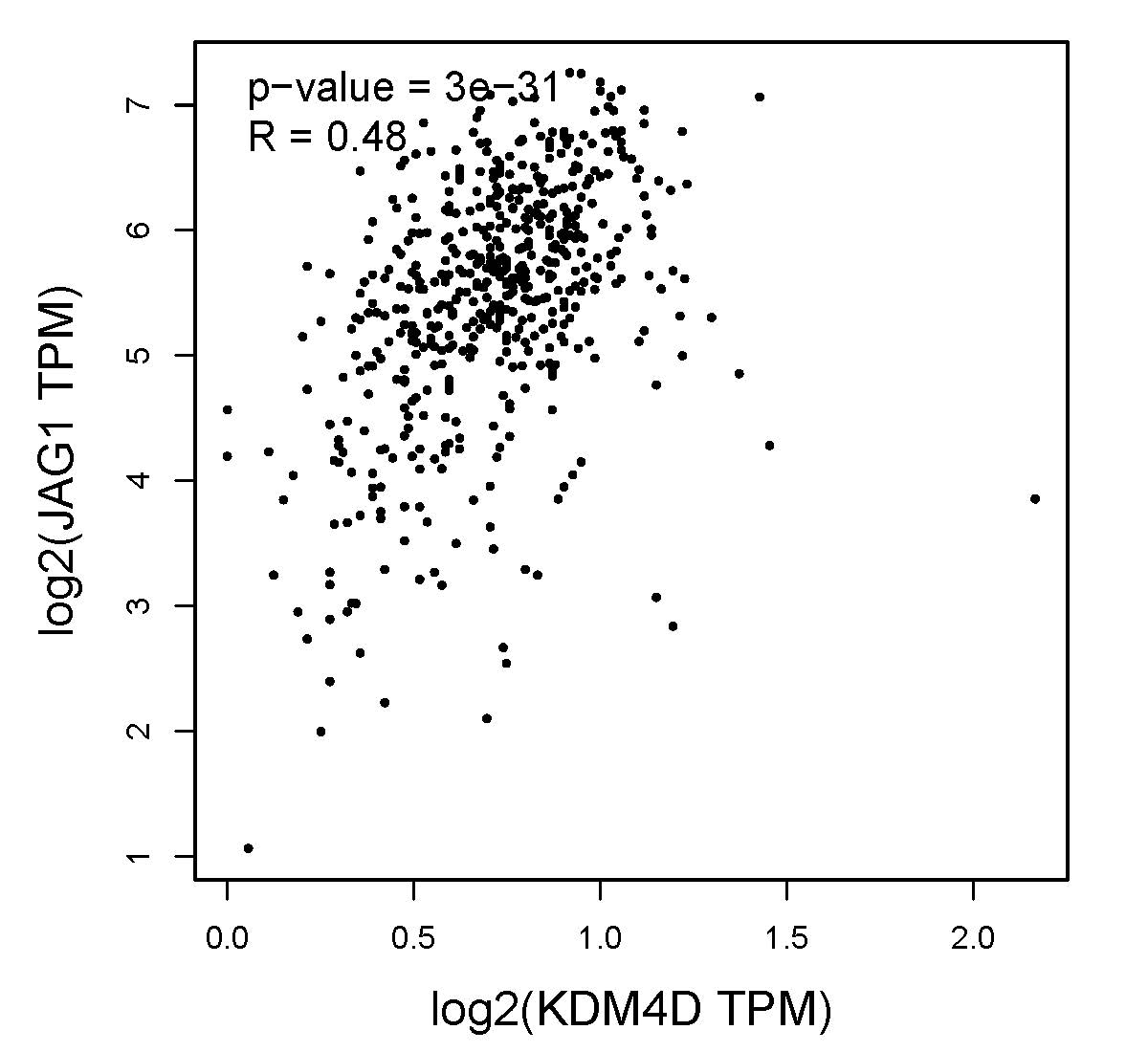
**Supplementary Figure 2** Correlation analysis demonstrated that JAG1 is positively associated with KDM4D.


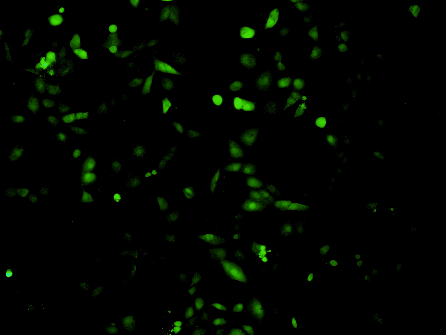


**Supplementary Figure 3** Representative images 786-O cells transfected with pcDNA-3.1(+)-JAG1, successfully transfected cells have eGFP green fluorescence.

**Supplementary Table 1 Primer sequences of quantitative real-time PCR**

| Gene | Sense primer (5'-3') | Anti-sense primer (5'-3') |
| --- | --- | --- |
| KDM4D | GGGCAGGGGTGTTTACTCAAT | TGTTTGCCAAATGGCGATACT |
| JAG1 | GTATCTGTCCACCTGGCTATGC | GTCACTGGCACGGTTGTAGC |
| STAT1 | ACTTTCCCTGACATCATTCGC | TCTACAGAGCCCACTATCCGAG |
| VEGFR3 | CAGCATCCTGACCATCCACA | ATGGGTCCTTTGAGCCACTC |
| AKT3 | TGGCACTCCAGAATATCTGGC | CTCCACCAAGGCGTTTATTTG |
| PLXND1 | CCATCAAGCAGCAAATCAACA | CCACTGGGAGTAGGGCACAT |
| TGFβ1 | CAGCAACAATTCCTGGCGATA | GCTAAGGCGAAAGCCCTCAAT |
| GADPH | CATCATCCCTGCCTCTACTGG | GTGGGTGTCGCTGTTGAAGTC |
